# A Study of Oral Health Parameters and the Properties and Composition of Saliva in Oncological Patients with and without Medication-Related Osteonecrosis of the Jaw Who Take Bisphosphonates

**DOI:** 10.3390/medicina59061073

**Published:** 2023-06-02

**Authors:** Hanna Sobczak-Jaskow, Barbara Kochańska, Barbara Drogoszewska

**Affiliations:** 1 Department of Maxillofacial Surgery, Medical University of Gdansk, 80-210 Gdansk, Poland; barbara.drogoszewska@gumed.edu.pl; 2 Department of Conservative Dentistry, Medical University of Gdansk, 80-210 Gdansk, Poland; barbara.kochanska@gumed.edu.pl

**Keywords:** biomarkers, bisphosphonates, caries, dental care, MRONJ, oral care, saliva, systemic diseases

## Abstract

*Background and Objectives*: The aim of this study was to examine how the status of the oral cavity, composition and properties of saliva change in oncological patients with and without Medication-Related Osteonecrosis of the Jaw (MRONJ) undergoing bisphosphonate therapy. *Materials and Methods*: A retrospective case–control study of 49 oncological patients using bisphosphonates (BPs) was conducted. The study population was divided into two groups—Group I consisted of 29 patients with MRONJ and Group II of 20 patients without MRONJ. The control group consisted of 32 persons without oncological history and without antiresorptive therapy. Standard dental examination included the assessment of the number of teeth remaining, teeth with caries and fillings, Approximal Plaque Index (API) and Bleeding on Probing (BOP). In terms of MRONJ, localization and stage were assessed. Laboratory tests of saliva included determination of pH and concentrations of Ca and PO_4_ ions, total protein, lactoferrin, lysozyme, sIgA, IgA, cortisol, neopterin, activity of amylase at rest, and stimulated saliva. The buffering capacity and microbiological tests (*Streptococcus mutans, Lactobacillus* spp. load) of stimulated saliva were also determined. *Results*: There were no statistically significant differences between the selected oral parameters and saliva of Group I and Group II. Significant differences were found between Group I and the control group. BOP, lysozyme and cortisol concentration were higher, while the number of teeth with fillings, Ca and neopterin concentrations were lower in comparison to the control group. In Group I, a significantly higher percentage of patients with a high colony count (>10^5^) of *Streptococcus mutans* and *Lactobacillus* spp. was also found. The significant differences between Group II and the control group concerned the concentrations of lysozyme, Ca ions, sIgA, neopterin and the colony count of *Lactobacillus* spp. In the Group I patients who received a significantly higher cumulative dose of BP compared to the Group II, a significant positive correlation was found between the received BP dose and the BOP. Most MRONJ foci were stage 2 and were mainly located in the mandible. *Conclusions*: Among oncological patients with and without MRONJ undergoing BP therapy compared to the control group, there are statistically significant differences in the dental, periodontal and microbiological status and in the composition of the saliva. Particularly noteworthy are the statistically significant differences in the decreased level of Ca ions, the increased level of cortisol and the elements of saliva related to the immune response (lysozyme, sIgA, neopterin). Additionally, a higher cumulative dose of BPs may affect the susceptibility to the development of osteonecrosis of the jaws. Patients undergoing antiresorptive therapy should receive multidisciplinary medical care, including dental care.

## 1. Introduction

Bisphosphonates (BPs) are potent inhibitors of bone resorption and are widely used in the treatment of patients with osteoporosis and bone metastases [1,2,3,4,5,6,7,8,9,10,11,12,13,14]. In cancer patients, BPs are used to prevent or reduce skeletal-related events [1,5,6]. In addition, these drugs are used in reducing the risk of bone loss associated with cancer treatment, in the treatment of multiple myeloma, hypercalcemia and bone pain in cancer patients [1,5,6,8,10,13,14].

Medication-Related Osteonecrosis of the Jaw (MRONJ) is one of the most serious complications of treatment with BPs and significantly contributes to a decrease in the quality of life of oncological patients [15]. Additionally, recurrent bone infections in the course of MRONJ can result in postponement or changes in oncological treatment and have negative effects on treatment outcome [16].

The current definition of MRONJ according to the American Association of Oral and Maxillofacial Surgeons (AAOMS) is based on all three of the following characteristics: (1) current treatment with antiresorptive (AR) medication (alone or with immune modulators) or antiangiogenic drugs, or a history of such treatment; (2) exposure of the jawbone or an intraoral/extraoral fistula lasting at least 8 weeks; (3) no history of jawbone radiotherapy or metastatic disease to the jaws [4].

So far, it has not been possible to clearly prove why osteonecrosis caused by AR treatment from all skeletal bones has a predilection to develop within the jaw bones. However, the literature lists several hypotheses for explaining this relationship. First, the suppression of bone metabolism caused by a high concentration of BPs in the bone contributes to necrosis [14]. These drugs tend to be deposited in high-metabolic bones, which include jaw bones, due to the presence of teeth and the ongoing remodeling of periodontal ligaments around their roots [14]. Therefore, large amounts of BPs accumulate in the alveolar ridge [17]. Second, the generalized toxic effects of BPs on epithelial cells have been documented [18,19,20]. The literature describes that BPs administered intravenously at clinically relevant doses impair the healing processes of the oral mucosa by inhibiting the proliferation of oral mucosa keratinocytes [19]. The jaw bones, covered with a thin layer of mucous membrane, are particularly susceptible to injuries during the use of dentures or during dental procedures [14,16]. The antiangiogenic properties of BPs also contribute to the deterioration of the healing process [21,22]. The resulting slower healing of the oral mucosa allows microorganisms to penetrate into underlying bone, facilitating secondary infection [13,17]. Moreover, Kos et al., in their studies on the colonization of bisphosphonate-coated hydroxyapatite discs by oral bacteria, confirmed that commonly used BPs can enhance adhesion and promote the formation of biofilms by a broad spectrum of bacteria [10]. Finally, the impaired immune response of the patient undergoing chemotherapy also promotes infection.

The condition of the oral cavity is reflected in the composition and properties of saliva. The components of saliva involved in the mechanisms of specific and non-specific immune response contribute to maintaining the oral cavity ecosystem in balance by protecting against the development of bacterial, viral and fungal infections [23,24,25]. In addition, saliva, due to its buffer system, prevents enamel demineralization and, due to the content of Ca and PO_4_ ions, enables its remineralization, contributing to the preservation of tooth integrity [24,25]. Saliva also performs functions related to food intake: it enables the perception of taste, is responsible for preparing a bite to be swallowed, and the amylase present in saliva is involved in the initial digestion of starch [23,24]. 

Decreased salivation has been reported in the literature in patients taking AR drugs [26]. A normal flow of saliva is necessary to ensure the lubrication of oral tissues [27]. Through its lubricating function, saliva protects the surface of the mucosa and improves oral cavity cleaning [23,24,25]. Due to the serious clinical consequences of MRONJ, a lot of effort has been put into the study of potential biomarkers, including those in saliva, that can help in assessing the risk of MRONJ. By analyzing the composition of saliva, the level of bone turnover markers was checked—in the case of *N*-terminal telopeptide of type I collagen (NTX), statistically significant differences in its level were observed in patients with MRONJ [28]. Significant differences were also noticed when examining the salivary proteome, where special attention was paid to the differences in the level of three proteins: matrix metalloproteinase-9 (MMP-9), alpha-1-antichymotripsin (AACT) and hemoglobin submit delta (HBD) [29,30]. These proteins determine the course of inflammatory reactions, bone remodeling and oxygen transport [29]. Additionally, the level of interleukin-6 (IL-6), which is a marker of the inflammatory reaction, is elevated in the saliva of patients diagnosed with MRONJ [31]. Analyzing the functioning of the oral immune system, as a result of the conducted tests, impairment of the function of neutrophils collected from the oral cavity was noted [32]. According to the position of the AAOMS from the Position Paper of 2022, due to low-quality evidence, no biomarkers are currently recommended to assess the risk of MRONJ, further research on their effectiveness is recommended [4].

The results of the research carried out so far and the observations of the authors of this study prompted further investigation. The authors hypothesize that in oncological patients taking BP, the properties and composition of saliva may be disturbed, which may result in unfavorable changes in the oral cavity ecosystem, deterioration of oral hygiene, and intensification of dental and periodontal diseases. This may be a potential cause of MRONJ. The aim of this study is to examine how the condition of the oral cavity as well as composition and properties of saliva change in oncological patients with and without MRONJ who have received BP treatment compared to the control group.

## 2. Materials and Methods

The study protocol was approved by the Ethics Committee of the Medical University of Gdansk, Poland (NKBBN/166/2016). Ethical aspects of this research were in accordance with the World Medical Association’s Declaration of Helsinki. 

This research is a part of a larger scientific project related to the changes in the oral health parameters, and properties and composition of saliva in patients undergoing antiresorptive therapy. This article is a continuation of a recently published study on the properties and composition of saliva in patients undergoing antiresorptive therapy for osteoporosis [33].

### 2.1. Patient Population

A total of 49 cancer patients taking AR drugs (31 women and 18 men) aged 41–84 years old (mean = 64.7 ± 11.1, Me = 67.0) were examined. All patients were treated with BPs administered intravenously as part of the oncology treatment protocol. 

The cancer patients were divided into two groups. Group I consisted of patients diagnosed with MRONJ. This disease was found in 29 patients (14 women and 15 men) aged 43–84 years old (mean = 67.3 ± 10.1, Me = 69.0). Group II consisted of 20 patients (17 women and 3 men) aged 41–81 years old (mean = 61.0 ± 11.7, Me = 61.5), in whom MRONJ was not diagnosed. The diagnosis of MRONJ was based on the AAOMS definition [3,4]. 

The control group consisted of 32 persons (18 women and 14 men) aged 51–83 years old (mean = 69.3 ± 6.9, Me = 69.0). In the case of the control group, patients with a history of oncological diseases and that are currently taking, or have in the past, AR drugs were excluded from this study. Furthermore, persons who took AR drugs for other reasons than oncological, e.g., osteoporosis, were also excluded. 

Participants with the following characteristics were all excluded from this study: patients with diseases of the salivary glands and/or active systemic infection, non-compliance with the rules of saliva collection and/or lack of consent to participate in this study.

The examination of the study participants as well as saliva collection were conducted at the Maxillofacial Surgery Clinic of the Medical University Hospital in Gdansk and at the Dental Surgery Department of a Medical Clinic in Gdynia. 

Table 1 presents the characteristics of Groups I and II in terms of oncological diagnosis.

Table 2 contains data on BPs used in Groups I and II, the total number of doses of BPs, and duration and the frequency of AR therapy. Both in Group I and Group II, the most commonly used BP was zoledronate (89.7% and 95.0%, respectively). The duration of BP therapy in both groups was similar, while the frequency of BP dosage and total number of BP doses were significantly higher in patients with MRONJ. The mean duration of therapy in Groups I and II was 18.5 ± 17.4 months (Me = 12.5, range 1.0–60.0).

Table 3 provides information on the prevalence of systemic risk factors for MRONJ among study participants. The information on chemotherapy and corticosteroid therapy relates to the treatment given during the oral and saliva examination, not the history of such treatment in the past. In Groups I and II, the prevalence of systemic MRONJ risk factors, with the exception of diabetes, was similar. There were also no statistically significant differences between both of the study groups and the control group in the incidence of diabetes and smoking.

### 2.2. Dental Examination

Each oncology patient participating in this study was examined for the presence of MRONJ symptoms. MRONJ staging was performed according to 2014 AAOMS guidelines, which are the following: at risk is defined as: “no apparent necrotic bone with no symptoms in patients who have been treated with oral or intravenous bisphosphonates”. Stage 0 is defined as: “no clinical evidence of necrotic bone but non-specific clinical findings, radiographic changes, and symptoms”. Stage 1 is defined as: “exposed and necrotic bone or a fistula that probes to bone in patients who are asymptomatic and have no evidence of infection”. Stage 2 is defined as: “exposed and necrotic bone or a fistula that probes to bone associated with infection as evidenced by pain and erythema in the region of exposed bone with or without purulent drainage”. Stage 3 is defined as: “exposed and necrotic bone or fistula that probes to bone in patients with pain, infection and ≥1 of the following: exposed and necrotic bone extending beyond the region of alveolar bone (i.e., inferior border and ramus in mandible, maxillary sinus, and zygoma in maxilla) resulting in pathologic fracture, extraoral fistula, oral antral or oral-nasal communication or osteolysis extending to inferior border of the mandible or sinus floor” [3]. 

Each participant underwent a standard dental examination, taking into account the condition of the dentition (number of retained teeth, number of teeth with caries—DT, number of teeth with fillings—FT), oral hygiene (Approximal Plaque Index), periodontal status (Bleeding on Probing). One dentist, a co-author of this article, performed the dental examination of all patients included in this study (H.S.-J.). In order to study oral hygiene, the Approximal Plaque Index (API) was used [34]. The presence of plaque in the interproximal spaces was examined using a probe. In quadrants 1 and 3, the presence of plaque on the proximal surfaces was examined from the palatal/lingual side while in quadrants 2 and 4 from the buccal side. The presence of dental plaque was considered a positive result. The percentage of sites with positive results was counted. The API ranges obtained from the examination are interpreted as follows: API = 70–100% corresponds to poor oral hygiene; API = 40–69% corresponds to average hygiene; API = 25–39% corresponds to reasonably good hygiene; API < 25% corresponds to optimal oral hygiene [35]. When evaluating the API, only places that formed a contact point were examined. API was not tested in the case of edentulism, the presence of wide interdental spaces (e.g., after a lost tooth), prosthetic bridge pontics and distal surfaces of the last teeth in the arch, which do not form a contact point with another tooth. Bleeding on Probing (BOP) was assessed for all retained teeth, at mesial, distal, buccal and lingual sites and it was recorded in a binary manner (presence/absence) [36]. The index was not calculated in edentulous patients. 

### 2.3. Saliva Collection

The material for this study was mixed saliva. A resting saliva sample and a stimulated saliva sample were collected from each study participant. Each participant received guidelines on the method of saliva collection. Saliva was collected in the morning. Each person participating in this study, two hours before saliva collection, was obliged to refrain from eating, drinking, mouthwash, tooth brushing, smoking and/or chewing gum. The spitting method was applied according to the standardized guidelines [37]. Resting saliva was collected by expectoration in the absence of chewing movements into a sterile Corning tube for five minutes. To obtain stimulated saliva, the actual saliva collection was preceded by chewing of a paraffin cube for one minute. Then, the stimulated saliva was released into the test tube for five minutes. Blood-contaminated saliva samples were not included in this study. Immediately after collection, the pH level was determined in resting saliva, and part of the stimulated saliva was used to test the pH level, buffer capacity and for microbiological tests. The obtained resting saliva and the rest of the stimulated saliva samples were frozen at −30 °C until the start of the biochemical tests. Laboratory tests of saliva were performed in the biochemistry laboratory of the Department of Conservative Dentistry of the Medical University of Gdansk [33].

### 2.4. Biochemical Analysis of Saliva

The pH level of the saliva was measured using Sigma-Aldrich pH Test Strips 4.5–10.0 (Sigma-Aldrich, St. Louis, MO, USA) [33]. 

The concentrations of Ca ions (mg%) and PO_4_ ions (mg%), were determined in the samples of non-centrifuged saliva. The Arsenanzo III method was used to determine the concentration of Ca ions in the examined saliva. This method uses the metallochromogen Arsenanzo III, which has a strong affinity for Ca ions and binds to them, which forms a colored complex. Absorbance is measured at a wavelength of 650 nm. The intensity of the color measured at 650 nm is proportional to the concentration of Ca in the sample. A reagent (Alpha Diagnostic sp. z o.o., Warsaw, Poland) containing the active ingredients Arsenanzo III and an imidazole buffer was used for the biochemistry test along with another Alpha Diagnostic reagent containing calcium, which was used for the standard curve. The reagent was added to the saliva sample at a ratio of 1:100 and the mixture was incubated for one minute. Absorbance was then measured at 650 nm and the Ca concentration was calculated from the standard curve [27,33]. The concentration of PO_4_ ions was tested using the direct method with phosphomolybdate based on the modified method of Daly and Ertingshausen. The reagent (Alpha Diagnostic sp. z o.o., Warsaw, Poland) was added to the saliva sample at a ratio of 1:100. After incubation for six minutes, absorbance was measured at a wavelength of 340 nm using a Sunrise f. TECAM spectrophotometer. The phosphorus concentration was calculated from the standard curve [33]. 

The tests of organic components were carried out in centrifuged saliva. The saliva was centrifuged for ten minutes at 14,500 rpm using an Eppendorf Mini Spin Plus centrifuge. The concentrations of total protein (mg/mL), lactoferrin (μg/mL), lysozyme (μg/mL, IgA (μg/mL), sIgA (μg/mL), neopterin (nmol/mL), cortisol (ng/mL) and amylase activity (U/mL) were determined in the collected saliva. The quantification of total protein in samples of resting and stimulated saliva was performed by the Lowry method [38]. This method uses the sensitive reaction of peptide bonds and tyrosine with Folin–Ciocâlteu reagent. As a result of the reaction, colored products are formed. The absorbance of the resulting color was read at a wavelength of 750 nm. The total protein concentration in the tested samples was calculated from the calibration curve. Measurements were carried out using the HACH LANGE DR 3900 spectrophotometer. The concentrations of lactoferrin, lysozyme, and immunoglobulin IgA were analyzed in saliva using the immunoenzymatic ELISA method. Non-commercial tests were used for this purpose. The immunoenzymatic ELISA method, with the use of commercially available tests, was also used to determine the concentration of sIgA immunoglobulin (Immuniq, Immunodiagnostik, Bensheim, Germany), the concentration of neopterin (Demeditec, Kiel, Germany), the concentration of cortisol (R&D, Minneapolis, MN, USA), and the activity of amylase (IBL, Hamburg, Germany). The absorbance was measured at a wavelength of 450 nm for commercial tests and 405 nm for non-commercial tests using a Sunrise f. TECAM spectrophotometer [33].

The CRT^®^ buffer tests by Ivoclar Vivadent (Ivoclar Vivadent, Schaan, Principality of Lichtenstein) were used to measure the buffering capacity of the saliva [33].

The CRT^®^ Bacteria tests by Ivoclar Vivadent (Ivoclar Vivadent, Schaan, Principality of Lichtenstein) were used to measure *Streptococcus mutans* (*S. mutans*) and *Lactobacillus* spp. load in the saliva. All saliva samples were incubated aerobically for 48 h at 37 °C. The number of bacterial colonies was read by comparing with reference cards provided by the manufacturer. The results are presented in the following ranges ˂ 10^5^ CFU/mL of saliva—low colony count; ≥10^5^ CFU/mL of saliva—high colony count.

### 2.5. Statistical Analysis

The statistical analyses were performed by using the statistical suite STATISTICA (data analysis software system), version 12.0 (StatSoft Inc., Tulsa, OK, USA) [33]. 

The Shapiro–Wilk W test was used to check whether the quantitative variable came from a normally distributed population. The Leven (Brown-Forsythe) test was used to test the hypothesis of equal variances.

The significance of differences between the two groups (model of unrelated variables) was tested by the tests of significance of differences: t-Student (or, in the absence of homogeneity of variance, the Welch test) or the Mann–Whitney U test (in the case of failure to meet the applicability conditions of the t-Student test or for variables measured on a ordinal). Significance of differences between more than two groups was checked by the F test (ANOVA) or the Kruskal–Wallis test (if the conditions of ANOVA applicability were not met). To obtain statistically significant differences between the groups, post hoc tests were used [33].

The independent chi-squared test was used for qualitative variables. In order to determine the relationship, strength, and direction between the variables, correlation analysis was used by calculating the Pearson’s and/or Spearman’s correlation coefficient [33]. 

The statistical significance level of α = 0.05 was used in all calculations [33].

## 3. Results

Table 4 presents the stage of MRONJ in Group I according to the AAOMS classification [3]. MRONJ stage 2 was most common in 17 (58.6%) patients. Less frequent were stage 1 (6 patients) and stage 3 (6 patients). No patient was diagnosed with MRONJ in stage 0. All patients in Group II were classified as at risk of MRONJ. Most often, changes in the nature of osteonecrosis of the jaw bones occurred after the extraction of one or more teeth. This affected 20 (69.0%) patients. However, in two patients (6.9%), MRONJ changes were caused by the use of an ill-fitted denture. One patient (3.4%) had Implant-Presence Triggered MRONJ. Additionally, in one patient (3.4%), the occurrence of MRONJ was associated with periodontal disease. The cause of MRONJ was unknown in 5 (17.2%) patients. MRONJ foci were most often located in the mandible and it concerned 21 (72.4%) patients. MRONJ lesions were less frequently located in the maxilla, where they were found in 7 (24.1%) patients. One patient (3.4%) had two foci, where one was in the maxilla and one in the mandible. Lesions were more common in the lateral sections of the jaw bones (80.0% of cases) than in the anterior section (20.0% of cases). Lesions in the lateral sections of the jaw bones on the right side were found in sixteen (53.3%) cases, and on the left side in eight (26.7%) cases.

Table 5 presents the results concerning the condition of the dentition and marginal periodontium as well as oral hygiene of the examined persons. The number of retained teeth was the lowest in patients from Group I (Me = 12) compared to Group II (Me = 20) and the control group (Me = 18.5), but these differences were not statistically significant. The number of teeth with active caries in Group I (Me = 1) and Group II (Me = 1.5), as well as in the control group (Me = 0) did not differ significantly. The number of teeth with fillings was the lowest in Group I (Me = 2) compared to Group II (Me = 9) and the control group (Me = 8). A statistically significant difference was found between Group I and the control group. The highest value of the BOP was found in patients from Group I (Me = 56%) compared to Group II (Me = 14%) and the control group (Me = 11%), with a statistically significant difference between Group I and the control group. The API, similarly to the BOP, was also the highest in Group I (Me = 100%), compared to Group II (Me = 71%) and the control group (Me = 69%), but the differences were not statistically significant.

As presented in Table 2, in Group I, the duration of BP therapy was on average 26.6 ± 16.4 months (Me = 24.0, range 6.0–60.0), while in Group II it was 20.5 ± 15.3 months (Me = 14.5, range 1.0–51.0). Group I patients received significantly more BP doses in the course of oncological treatment compared to Group II. Patients in Group I received an average of 26.6 ± 16.4 (Me = 24.0, range 6.0–60.0) doses and patients in Group II received an average of 14.1 ± 16.4 (Me = 7.5, range 1.0–51.0) doses. In Group I, a significant positive correlation was found between the number of doses of BP and BOP (r = 0.4545). In Group II, a significant positive correlation was found between the number of doses taken and the concentration of cortisol in stimulated saliva (r = 0.5094). 

As can be seen from the data presented in Table 6 and Table 7, no statistically significant differences were found between the examined parameters of saliva in Group I compared to Group II.

The data presented in Table 6 and Table 7 indicate statistically significant differences in the unstimulated (US) and stimulated (SS) saliva composition of patients in Group I and the control group. Lower concentrations of Ca ions (US: Me = 4.24 mg%, range 2.00 mg%–13.12 mg%; SS: Me = 3.28; range 1.68 mg%–11.39 mg%) were found in Group I compared to the control group (US: Me = 5.41 mg%, range 3.07 mg%–15.90 mg%; SS: Me = 4.51 mg%, range 1.02 mg%–14.18 mg%), but this difference was statistically significant only for resting saliva. Analyzing organic components associated with innate immunity, it was found that the concentration of lysozyme in both resting and stimulated saliva (US: Me = 1.32 μg/mL, range 0.10 μg/mL–7.66 μg/mL; SS: Me = 1.85 μg/mL, range 0.21 μg/mL–9.35 μg/mL) was significantly higher in Group I compared to the control group (US: Me = 0.30 μg/mL range 0.03 μg/mL–0.72 μg/mL; SS: Me = 0.35 μg/mL, range 0.04 μg/mL–0.72 μg/mL). The concentration of neopterin in stimulated saliva was statistically significantly lower in patients in Group I (Me = 3.78 mmol/L, range 1.01 mmol/L–43.26 mmol/L) compared to the control group (Me = 11.55 mmol/L, range 1.46 mmol/L–39.23 mmol/L). The concentration of neopterin in the resting saliva (Me = 4.07 mmol/L, range 1.13 mmol/L–109.76 mmol/L) was also lower than in the control group (Me = 10.34 mmol/L, range = 1.35 mmol/L–124.30 mmol/L), but the difference was not statistically significant. The level of cortisol in resting and stimulated saliva (US: Me = 5.46 ng/mL, range 0.97 ng/mL–43.00 ng/mL; SS: Me = 6.51 ng/mL, range 0.89 ng/mL–43.00 ng/mL) was higher in Group I compared to the control group (US: Me = 2.99 ng/mL, range 0.84 ng/mL–6.71 ng/mL; SS: Me = 3.21 ng/mL, range = 0.75 ng/mL–14.20 ng/mL), but the difference was statistically significant only for resting saliva. There were no statistically significant differences between Group I and the control group in terms of: pH level, amylase activity, IgA and sIgA levels, concentration of PO_4_ ions, total protein, lactoferrin in resting and stimulated saliva.

Significant differences occurred between some parameters of resting and stimulated saliva in people in Group II and the control group. In the case of inorganic components, it was found that the concentration of Ca ions was statistically significantly lower both in the resting and stimulated saliva (US: Me = 3.07 mg%, range 1.02–9.67 mg%; SS: Me = 2.83 mg%, range 0.73 mg%–9.30 mg%) of patients in Group II compared to the control group (US: Me = 5.41 mg%, range 3.07 mg%–15.90 mg%; SS: Me = 4.51 mg%, range 1.02 mg%–14.18 mg%). In Group II a statistically significant increase in the concentration of lysozyme was found in both resting and stimulated saliva (US: Me = 1.43 μg/mL, range 0.22 μg/mL–7.48 μg/mL; SS: Me = 1.32 μg/mL, range 0.22 μg/mL–5.70 μg/mL) compared to the control group (US: Me = 0.30 μg/mL, range 0.03 μg/mL–0.72 μg/mL; SS: Me = 0.35 μg/mL, range 0.04 μg/mL–0.72 μg/mL). Analyzing the parameters of specific immunity in resting and stimulated saliva, it was found that the concentration of sIgA (US: Me = 313.47 μg/mL, range 96.70 μg/mL–555.55 μg/mL; SS: Me = 99.03 μg/mL, range 40.00 μg/mL–1898.32 μg/mL) was significantly lower in Group II compared to the control group (US: Me = 529.03 μg/mL, range 96.67 μg/mL–1193.54 μg/mL; SS: Me = 380.26 μg/mL, range 66.60 μg/mL–1212.90 μg/mL). The concentration of neopterin in Group II (US: Me = 3.79 nmol/L, range 1.09 nmol/L–155.40 nmol/L; SS: Me = 3.82 nmol/L, range 1.22 nmol/L–13.44 nmol/L) was also lower than in the control group (US: Me = 10.34 nmol/L, range 1.35 nmol/L–124.30 nmol/L; SS: Me = 11.55 nmol/L, range 1.46 nmol/L–39.23 nmol/L), but this difference was statistically significant only in the case of stimulated saliva. There were no statistically significant differences between Group II and the control group in the case of pH, amylase activity, cortisol, concentration of PO_4_ ions, total protein, lactoferrin, IgA in resting and stimulated saliva.

The buffering capacity of saliva in patients treated with BPs, regardless of the occurrence of MRONJ, and in the control group, was similar (Table 8).

The results of microbiological tests of saliva indicate significant differences in the number of colonies of *S. mutans* and *Lactobacillus* spp. in patients in Groups I and II and in the control group (Table 9). For *S. mutans*, the percentage of individuals with a high colony count (>10^5^) was found to be highest in Group I (34.5%), lower in Group II (10.5%) and lowest in the control group (3.3%). There was a statistical difference between Group I and the control group. Additionally, in the case of *Lactobacillus* spp., it was found that the percentage of individuals with a high colony count (>10^5^) was highest in Group I (51.7%), lower in Group II (26.3%), and lowest in the control group (6.5%). Statistically significant differences were found between Group I and Group II and the control group.

## 4. Discussion

Not only topical drugs, but also many groups of systemic drugs affect the condition of the oral cavity and the composition and properties of saliva [39]. Cancer patients are particularly at risk of oral complications during oncological treatment [39,40]. This relationship is primarily related to the multitude of drugs used in cancer therapy, which, due to the lack of specificity in action, may have an adverse effect on the condition of the oral cavity [39]. In addition, the poor condition of teeth and neglect of oral hygiene in this group of patients may contribute to the exacerbation of complications of oncological treatment in the oral cavity. The most common oral complications among cancer patients include mucositis, xerostomia and dysphagia [40]. MRONJ is a rare complication. Its occurrence is estimated at less than 5% in the population of cancer patients treated with zoledronate [4]. However, the consequences of this disease are serious, long-term and may require surgical treatment. Despite many efforts devoted to research on the pathogenesis of MRONJ, so far it has not been fully explored. Risk factors for MRONJ associated with AR treatment include long-term use of BPs [3,4,14,16]. Symptoms of jaw osteonecrosis develop significantly more often in patients taking intravenous BPs for oncological indications [1,3,4,11,14,16,41]. Patients using zoledronate and pamidronate are at particular risk of developing the disease [1,3,4,11,14,16,41]. In our study, patients with symptoms of MRONJ received a significantly higher cumulative dose of BPs compared to cancer patients without MRONJ. A similar relationship was noted by Kos et al. and Carmagnola et al., among oncological patients taking AR drugs with and without MRONJ [11,12]. This relationship may be related to the mechanism of action of BPs, which incorporate with the bone matrix for a long period of time, which is up to ten years, effectively inhibiting bone resorption [14]. Comorbid risk factors associated with the development of MRONJ include chemotherapy, use of corticosteroids, diabetes and smoking [3,4,7,16]. In Group I, a significantly higher percentage of patients with diabetes was found compared to Group II. Among the analyzed risk factors, the presence of local infection within the oral cavity plays an important role in pathogenesis of MRONJ [7]. In the literature, it is reported that the local factors contributing to osteonecrosis of the jaw bones include dental surgery procedures involving the exposure of the alveolar bone, of which tooth extraction is the most common procedure [3,4,7,14,16]. It has been speculated that the main risk factor is actually an odontogenic inflammation, which is an indication for tooth extraction [7]. The use of ill-fitted dentures, may also predispose to mucosal injuries, which are the gateway to bone infection [14,16]. Other mentioned in the literature local risk factors include periodontal disease, periimplantitis and poor oral hygiene [3,4,9,11,16].

Kos et al. and Carmagnola et al. analyzed the dental and periodontal status in patients with MRONJ [11,12]. In the study conducted by Kos et al., the value of DMFT index (D—decayed, M—missing, F—filled, and T—teeth) was significantly higher in the group of patients with MRONJ compared to the group of patients undergoing AR therapy without osteonecrosis [11]. In the studies of Carmagnola et al., no statistically significant differences in DMFT were confirmed between the group of patients taking AR drugs with and without MRONJ [12]. In our study a significantly lower number of fillings was found in the Group I compared to the control group. However, the result may be affected by the small number of retained teeth in this group of patients among the compared groups. Another indicator that showed a statistically significant difference between the group of patients with MRONJ compared to the control group was the BOP rate [36]. Longer exposure to BPs was also associated with a higher BOP index, as evidenced by the positive correlation in Group I. Significantly higher BOP values in Group I (Me = 56%) indicate a greater extent of gingival inflammation in this population of patients. Gingivitis may be a symptom of periodontal disease or predisposition to it, especially with poor oral hygiene, as indicated by the results of API studies [42]. In Group I, the median of this parameter was 100%. Similarly, Kos et al., obtained a poorer oral hygiene in the group of patients with MRONJ compared to the control group, which was constituted of a group of oncological patients subjected to BPs therapy without MRONJ [11]. These results, however, were statistically insignificant. In the same study, in the group of MRONJ patients they confirmed more advanced periodontal disease compared to the group without MRONJ [11]. In that case, results were statistically significant. However, Carmagnola et al., who also examined periodontal status, found no statistically significant differences between the group of patients treated with BPs with and without MRONJ [12]. Inflammations of the periodontium and periapical area are often indications for tooth extraction, which is the event initiating osteonecrosis [3,4,7,14,16]. However, as recent studies indicate, periodontal disease itself may be a trigger for MRONJ [9]. Therefore, procedures aimed at plaque, calculus and caries should be implemented in the group of patients at risk of MRONJ.

When analyzing the location of MRONJ lesions, most of the foci of necrosis affected the mandible with a predilection to the lateral sections of the jaw bones, which is consistent with the cases reported so far in the literature [14,16]. According to Otto et al., the higher incidence of necrosis in the mandible may, as in bacterial osteitis, be associated with a significantly reduced collateral vascularity and a different bone structure compared to the jaw—a higher ratio of cortical bone to cancellous bone [7]. The lateral section of the jaw bones is, in turn, more prone to necrotic changes due to the higher frequency of inflammatory periapical and marginal periodontal lesions affecting the posterior teeth [7]. Moreover, this area is more difficult to clean manually, which results in a poorer level of hygiene in this area.

Analyzing the composition of saliva, among inorganic elements, the level of Ca ions showed statistically significant differences. The concentration of Ca ions was significantly lower in the resting saliva of patients from Group I and in the resting and stimulated saliva of Group II compared to the control group. The ability of BPs to inhibit bone resorption affects calcium metabolism in the body [2]. As a result of the use of BPs, the level of calcium in the blood serum decreases [2]. Hence, intravenous BPs have been used in the treatment of hypercalcemia, which is a serious metabolic disorder in oncological patients [8]. A decrease in the concentration of calcium ions in saliva, as well as in blood, may also be due to the effectiveness of BP therapy. An analogous relationship was noted in the study of patients undergoing AR therapy due to osteoporosis, where a decrease in the concentration of calcium ions was found in both resting and stimulated saliva, but the relationship was statistically significant only for stimulated saliva [33]. BPs also show the ability to inhibit the calcification process [43]. This property has become the basis for the topical use of BPs as an additive in the composition of toothpastes, in order to reduce the ability to form tartar on the tooth surface [44]. Generally used BPs are detected in saliva [45]. Therefore, these drugs may also potentially inhibit the mineralization of tartar by a direct mechanism. An interesting study was conducted by Sun et al., who studied in vitro the topical effect of etidronate, a drug belonging to the group of BPs, on the enamel. They noticed that the affinity of etidronate to hydroxyapatite crystals and its deep penetration into the enamel structure contributes to a long-term arrest of caries progression [46]. However, research by Tuncer et al. on animal models indicates that pamidronate administered generally during the growth and development stage causes malformations of enamel and dentin and interferes with tooth eruption and mandibular growth in newborn rats [47]. Inorganic ions Ca and PO_4_ play an important role in the remineralization processes of tooth hard tissues [24,48]. The processes of demineralization and remineralization of tooth hard tissues are a continuous process, and their imbalance results in enamel erosion and may lead to the development of caries. In the conducted study, a statistically significantly lower level of calcium ions was found in the saliva of patients from Groups I and II compared to the control group, which may lead to an increased risk of developing enamel demineralization processes and reduced tartar mineralization in these groups of patients.

In our study, there are statistically significant differences in the salivary components related to the immunity of the saliva, i.e., lysozyme and sIgA, are particularly noteworthy. The problem of innate and acquired immune disorders is increasingly discussed in the literature as a factor predisposing to the development of MRONJ [4,49]. The elements of oral non-specific immunity include lysozyme [24]. This protein has the properties of an enzyme that leads to the hydrolysis of the polysaccharide layer of the Gram-positive bacterial cell wall [23,24]. Immunoglobulins responsible for specific immunity work in cooperation with the mechanisms of non-specific immunity. The most abundant immunoglobulin in saliva is sIgA, whose function is to bind to antigens in the saliva, in the oral mucosa and in the acquired enamel pellicle [24,25]. In addition, sIgA inhibits the adhesion of microorganisms to the surface of the oral mucosa and teeth and contributes to the elimination of bacteria from the oral cavity by agglutination, including *S. mutans* [24]. Sun et al., in a study on the oral mucosal immune status in patients with malignant tumors, assessed the level of lysozyme and sIgA in saliva [50]. The listed salivary components were tested only in resting saliva. In the population of oncological patients, compared to the control group, Sun et al., obtained significantly lower levels of sIgA and significantly higher levels of lysozyme in saliva [50]. In our study, similar results were obtained for the examined saliva components, except for Group I, where the decrease in sIgA level was not statistically significant. Impairment of mucosal immunity, for which sIgA is responsible, is of critical importance in the population of cancer patients due to the risk of infections caused by host-borne pathogens. This group of pathogens includes bacteria of the genus *Actinomyces* spp., which constitute the saprophytic flora of the oral cavity, and under physiological conditions do not penetrate the border of a healthy mucosa. However, in the event of a decrease in immunity combined with trauma to the oral mucosa, *Actinomyces* spp. microorganisms may show pathogenic activity. *Actinomyces* spp. infection is particularly often detected in clinical MRONJ lesions [3,12,13,41]. In addition, the immune system is involved in tissue repair and supports bone remodeling [49]. Disturbances in both of these processes have a basis in the pathogenesis of MRONJ [4,49]. The obtained results in the field of oral immunity parameters, on the one hand, prove the activation of the non-specific response in the form of lysozyme among oncological patients taking BPs regardless of MRONJ, and on the other hand, the reduction in the specific response among oncological patients treated with BPs without MRONJ. Dysregulations of immune mechanisms may potentially contribute to the development of MRONJ through the development of infections, impaired healing processes of soft tissues and bones.

In the conducted study, statistically significant differences were noted in the concentrations of neopterin, which is an indicator of the cellular immune response in humans [51,52]. It is secreted by monocytes/macrophages and dendritic cells as a result of stimulation by interferon gamma (INF-γ), whose source is T lymphocytes [51,52,53]. The determination of neopterin has found application in clinical practice as a reflection of the activation of the cellular response. The level of neopterin is sometimes determined in the course of bacterial, viral, neurological and cardiovascular diseases [52]. In addition, an increase in the value of this indicator was observed in the course of some cancers [52,53]. The incidence of elevated values of this indicator varies depending on type of cancer, in the case of multiple myeloma elevated neopterin values at the time of diagnosis of the disease are observed in 60–80% of patients, and in the case of breast and prostate cancer only in 20–40% [52,53]. Among cancer patients with symptoms of anemia, fatigue, depression, weight-loss and cachexia, elevated values of this indicator are also found [53]. The concentration of neopterin is reduced when there is a good response to oncological treatment and remission of the disease [52]. Taking into account the condition of the oral cavity, neopterin has also found application in the diagnosis of periodontal diseases [54,55]. When examining the concentration of neopterin in the saliva and urine of patients with periodontal disease, Vrecko et al. found a positive correlation and a statistically significant increase in the level of the indicator in saliva with the greater number of inflamed teeth [55]. These results are consistent with the results of the study by Mahendra et al., who, examining 20 patients with chronic periodontal disease, noted a significantly higher concentration of neopterin in the saliva of this group of patients compared to the healthy subjects [54]. The results of studies by Vrecko et al. and Mahendra et al. suggest that neopterin contained in saliva may also potentially be a marker of inflammation in the case of local infections within the oral cavity. Further research in this direction is required. In our research, the level of neopterin was significantly lower in the stimulated saliva of Groups I and II compared to the control group. These results indicate a reduced cellular immunity among oncological patients treated with BPs, which may favor the development of local infections and pathological conditions within the oral cavity.

In our study, significantly higher levels of cortisol were found in the resting saliva of cancer patients with symptoms of MRONJ. However, in the stimulated saliva of Group II, a positive correlation was found between the level of cortisol and the cumulative dose of BPs. Cortisol is a steroid hormone secreted in response to stress [56,57]. Cancer is associated with a change in a person’s life situation, which exposes the patient to significant stress during its course. This is confirmed by the results of Lambert et al., who, in their study on breast cancer survivors, describe disturbances in the secretion of cortisol in a group of oncological patients [56]. As proved by Sephton et al., dysregulation of cortisol secretion in cancer patients is also associated with impaired cell-mediated immunity [58]. This relationship is confirmed in our study by the reduced concentrations of neopterin in the stimulated saliva of patients from Groups I and II compared to the control group. In addition, in the literature, elevated cortisol levels are described in the course of numerous diseases of the oral cavity, such as caries, periodontal disease or lichen planus [57,59,60]. In the results of our study, the level of cortisol was significantly higher in the group of cancer patients with MRONJ compared to the control group, but this difference was statistically significant only in the case of resting saliva. Elevated cortisol levels may be associated not only with the course of the underlying disease—cancer—but also with the presence of oral infections in this group of patients. However, in order to confirm this hypothesis, further research on the oral cavity condition in patients undergoing BPs therapy is required.

In the conducted study, microbiological tests of stimulated saliva were performed; and in terms of the number of *S. mutans* and *Lactobacillus* spp. colonies, statistically significant differences have been noted between the groups. Summing up the results of microbiological tests of stimulated saliva of patients taking BPs with and without MRONJ, it can be concluded that the examined patients experienced significant disturbances in the oral cavity ecosystem with respect to *S. mutans* and *Lactobacillus* spp. bacteria, which are important in terms of caries development.

The limitations of our study include the small number of patients. Moreover, chemotherapy used in the course of oncological treatment may contribute to the development of pathological changes in the oral cavity and affect results of saliva tests. Patients participating in this study were subjected to various oncological treatment regimens depending on the type of cancer. Some medications used in the course of such oncological treatment may also increase the risk of developing MRONJ. The group of drugs that constitute an additional risk factor for MRONJ includes corticosteroids and antiangiogenic drugs, which are routinely used in chemotherapy regimens of multiple myeloma and breast cancer. The general health of patients related to diseases they suffer from, such as diabetes, can also affect the condition of the oral cavity and the components of saliva. Despite our study’s limitations, the results of the conducted research confirm the basic assumption of the hypothesis. Further research should increase the size of the study groups and select for a more unified patient population.

## 5. Conclusions

1. Among oncological patients with and without MRONJ undergoing bisphosphonate (BP) therapy compared to the control group, there are statistically significant differences in the dental, periodontal and microbiological status and in the composition of the saliva.
2. Particularly noteworthy are the statistically significant differences in the decreased level of Ca ions, the increased level of cortisol and the elements of saliva related to the immune response (lysozyme, sIgA, neopterin). Saliva-related immunological factors may have an impact on oral health, including potential for the development of MRONJ. Further research in this direction is indicated.
3. In the group of patients with MRONJ who received a significantly higher cumulative dose of BP compared to the group without MRONJ, a significant positive correlation was found between the received BP dose and the Bleeding on Probing (BOP) index.
4. Patients undergoing antiresorptive therapy should receive multidisciplinary medical care, including dental care.

## Figures and Tables

**Table 1:** Distribution of malignant diseases in patients taking bisphosphonates (Group I—patients with MRONJ; Group II—patients without MRONJ).

Study Groups	Breast Cancer	Prostate Cancer	Multiple Myeloma	Kidney Cancer
Group I (*n* = 29)	10 (34.5%)	12 (41.4%)	6 (20.7%)	1 (3.5%)
Group II (*n* = 20)	12 (60.0%)	1 (5.0%)	6 (30.0%)	1 (5.0%)

**Table 2:** Bisphosphonate use and duration of therapy in Group I (patients with MRONJ) and Group II (patients without MRONJ).

Parameter		Group I *n* = 29 (100%)	Group II *n* = 20 (100%)	*p*-Value
Type of BP	Z	26 (89.7%)	19 (95.0%)	0.990 ^1^
	P	1 (3.4%)	0 (0.0%)	
	P + Z	2 (6.9%)	1 (5.0%)	
Length of BP therapy (months)	Mean ± SD	26.6 ± 16.4	20.5 ± 15.3	0.272 ^2^
	Range	6.0–60.0	1.0–51.0	
	Me	24.0	14.5	
Frequency of BP dosage (weeks)	Mean ± SD	4.0 ± 0.0	11.7 ± 10.5	0.040 ^2,^*
	Range	4.0–4.0	4.0–26.0	
	Me	4.0	4.0	
Total number of BP doses during oncological treatment	Mean ± SD	26.6 ± 16.4	14.1 ± 16.4	0.003 ^2,^*
	Range	6.0–60.0	1.0–51.0	
	Me	24.0	7.5	

Legend: ^1^ Chi-square test, ^2^ U Mann–Whitney test, * α = 0.05, SD—standard deviation, MRONJ—Medication-Related Osteonecrosis of the Jaw, BP—bisphosphonate, Z—zoledronate, P—pamidronate, and P + Z—pamidronate + zoledronate.

**Table 3:** Characteristics of study groups and the control group in terms of systemic MRONJ risk factors.

Risk Factor	Group I *n* = 29 (100%)	Group II *n* = 20 (100%)	Control Group *n* = 32 (100%)	*p*-Value
Chemotherapy	8 (27.6%) ^a^	7 (35.0%) ^b^	0 (0.0%) ^c^	^a–b^ 0.662 ^1^
				^a–c^ 0.01 ^1,^*
				^b–c^ 0.01 ^1,^*
Corticosteroid intake	4 (13.8%) ^a^	5 (25.0%) ^b^	0 (0.0%) ^c^	^a–b^ 0.320 ^1^
				^a–c^ 0.030 ^1,^*
				^b–c^ 0.003 ^1,^*
Smoking habits	6 (20.7%) ^a^	2 (10.0%) ^b^	2 (6.3%) ^c^	^a–b^ 0.320 ^1^
				^a–c^ 0.095 ^1^
				^b–c^ 0.622 ^1^
Diabetes	7 (24.1%) ^a^	0 (0.0%) ^b^	4 (12.5%) ^c^	^a–b^ 0.012 ^1,^*
				^a–c^ 0.239 ^1^
				^b–c^ 0.100 ^1^

Legend: ^1^ Chi-square test, * α = 0.05, indication of significant differences between the specified parameters: ^a–b^, ^a–c^, ^b–c^.

**Table 4:** Assessment of Medication-Related Osteonecrosis of the Jaw (MRONJ) staging according to the 2014 AAOMS guidelines in subjects taking bisphosphonates (BPs) in Group I and Group II (Group I—patients with MRONJ; Group II—patients without MRONJ).

Study Groups	Severity of MRONJ in Subjects Taking BPs				
	At Risk	Stage 0	Stage 1	Stage 2	Stage 3
Group I *n* = 29 (100%)	0 (0.0%)	0 (0.0%)	6 (20.7%)	17 (58.6%)	6 (20.7%)
Group II *n* = 20 (100%)	20 (100%)	0 (0.0%)	0 (0.0%)	0 (0.0%)	0 (0.0%)

**Table 5:** Oral health characteristics of study groups and the control group.

Oral Health Indicators	Study Groups: Oncological Patients Treated with Bisphosphonates			
	with MRONJ (Group I) (*n* = 29)	without MRONJ (Group II) (*n* = 20)	Control Group (*n* = 32)	*p*-Value
Number of decayed teeth				0.373 ^1^
Range	0.0–9.0	0.0–12.0	0.0–6.0	
Me	1.0	1.5	0.0	
Number of filled teeth				0.031 ^1,^*
Range	0.0–20.0	0.0–22.0	0.0–21.0	^a–c^ 0.036 ^2,^*
Me	2.0 ^a^	9.0	8.0 ^c^	
Number of retained teeth				0.074 ^1^
Range	0.0–32.0	0.0–28.0	0.0–29.0	
Me	12.0	20.0	18.5	
Approximal Plaque Index API (%)	*n* = 23	*n* = 17	*n* = 30	0.077 ^1^
Range	38.0–100.0	25.0–100.0	31.0–100.0	
Me	100.0	71.0	69.0	
Bleeding on Probing BOP (%)	*n* = 24	*n* = 17	*n* = 31	0.004 ^1,^*
Range	0.0–100.0	3.0–100.0	0.0–100.0	^a–c^ 0.004 ^2,^*
Me	56.0 ^a^	14.0	11.0 ^c^	

Legend: MRONJ—Medication-Related Osteonecrosis of the Jaw, ^1^ the Kruskal–Wallis test, ^2^ Post hoc test, * α = 0.05, Me—median, indication of significant differences between the specified parameters: ^a–c^, *n*—the number of study participants in whom it was possible to measure a given indicator (the indices impossible to calculate in the case of edentulism, and in the case of API also in the absence of a contact point between teeth).

**Table 6:** Comparison of biochemical parameters in unstimulated saliva from study groups and the control group.

Unstimulated Saliva (US)	Study Groups: Oncological Patients Treated with BPs			
	with MRONJ (Group I) (*n* = 29)	without MRONJ (Group II) (*n* = 20)	Control Group (*n* = 32)	*p*-Value
pH	*n* = 28	*n* = 20	*n* = 32	0.221 ^1^
Range	5.0–8.0	5.0–7.5	5.5–8.0	
Me	6.5	6.5	7.0	
Ca (mg%)	*n* = 28	*n* = 19	*n* = 32	0.001 ^1,^*
Range	2.00–13.12	1.02–9.67	3.07–15.90	^a–c^ 0.050 ^2,^*
Me	4.24 ^a^	3.07 ^b^	5.41 ^c^	^b–c^ 0.001 ^2,^*
PO4 (mg%)	*n* = 28	*n* = 18	*n* = 32	0.136 ^1^
Range	7.90–106.46	7.71–50.36	5.15–66.62	
Me	19.07	20.60	14.62	
Total Protein (mg/mL)	*n* = 28	*n* = 19	*n* = 28	0.783 ^1^
Range	0.64–5.95	1.13–4.12	0.42–6.07	
Me	2.03	2.14	2.35	
Lactoferrin (μg/mL)	*n* = 27	*n* = 20	*n* = 28	0.564 ^1^
Range	2.20–117.00	5.00–166.00	1.48–230.00	
Me	16.04	21.75	24.23	
Lysozyme (μg/mL)	*n* = 28	*n* = 19	*n* = 28	<0.001 ^1,^*
Range	0.10–7.66	0.22–7.48	0.03–0.72	^a–c^ <0.001 ^2,^*
Me	1.32 ^a^	1.43 ^b^	0.30 ^c^	^b–c^ 0.001 ^2,^*
sIgA (μg/mL)	*n* = 28	*n* = 14	*n* = 29	0.042 ^1,^*
Range	2.71–2781.03	96.70–555.55	96.67–1193.54	^b–c^ 0.008 ^2,^*
Me	363.10	313.47 ^b^	529.03 ^c^	
IgA (μg/mL)	*n* = 28	*n* = 19	*n* = 27	0.191 ^1^
Range	24.00–1279.23	42.74–1419.90	38.88–1125.39	
Me	274.61	415.79	223.69	
Cortisol (ng/mL)	*n* = 28	*n* = 18	*n* = 32	0.005 ^1,^*
Range	0.97–43.00	0.98–40.00	0.84–6.71	^a–c^ 0.005 ^2,^*
Me	5.46 ^a^	5.22	2.99 ^c^	
Neopterin (nmol/l)	*n* = 26	*n* = 16	*n* = 31	0.102 ^1^
Range	1.13–109.76	1.09–155.40	1.35–124.30	
Me	4.07	3.79	10.34	
Amylase (U/mL)	*n* = 27	*n* = 14	*n* = 29	0.409 ^1^
Range	12.68–465.50	10.63–442.60	12.15–538.57	
Me	77.80	158.70	84.99	

Legend: ^1^ The Kruskal–Wallis test, ^2^ post hoc test, * α = 0.05, Me—median, indication of significant differences between the specified parameters: ^a–c^, ^b–c^, *n*—the number of saliva samples in which the test was performed (in some cases, the amount of saliva collected was not sufficient to perform the test).

**Table 7:** Comparison of biochemical parameters in stimulated saliva from study groups and the control group.

Stimulated Saliva (SS)	Study Groups: Oncological Patients Treated with BPs			
	with MRONJ (Group I) (*n* = 29)	without MRONJ (Group II) (*n* = 20)	Control Group (*n* = 32)	*p*-Value
pH	*n* = 29	*n* = 20	*n* = 32	0.155 ^1^
Range	5.5–8.0	5.5–8.0	5.5–8.0	
Me	7.5	7.0	7.5	
Ca (mg%)	*n* = 28	*n* = 19	*n* = 32	0.012 ^1,^*
Range	1.68–11.39	0.73–9.30	1.02–14.18	^b–c^ 0.011 ^2,^*
Me	3.28	2.83 ^b^	4.51 ^c^	
PO4 (mg%)	*n* = 28	*n* = 19	*n* = 32	0.389 ^1^
Range	6.84–50.36	6.84–49.63	5.85–52.19	
Me	14.56	15.04	11.90	
Total Protein (mg/mL)	*n* = 28	*n* = 19	*n* = 31	0.840 ^1^
Range	0.57–4.60	0.96–4.15	0.39–6.02	
Me	1.48	1.45	1.83	
Lactoferrin (μg/mL)	*n* = 28	*n* = 16	*n* = 29	0.538 ^1^
Range	1.48–120.00	0.20–82.00	0.72–35.60	
Me	18.54	14.60	17.73	
Lysozyme (μg/mL)	*n* = 27	*n* = 19	*n* = 29	<0.001 ^1,^*
Range	0.21–9.35	0.22–5.70	0.04–0.72	^a–c^ <0.001 ^2,^*
Me	1.85 ^a^	1.32 ^b^	0.35 ^c^	^b–c^ 0.001 ^2,^*
sIgA (μg/mL)	*n* = 27	*n* = 17	*n* = 30	0.004 ^1,^*
Range	14.79–2716.42	40.00–1898.32	66.60–1212.90	^b–c^ 0.003 ^2,^*
Me	200.00	99.03 ^b^	380.26 ^c^	
IgA (μg/mL)	*n* = 28	*n* = 19	*n* = 30	0.102 ^1^
Range	22.00–1463.69	27.88–1014.41	22.31–1499.29	
Me	259.63	266.50	130.33	
Cortisol (ng/mL)	*n* = 27	*n* = 19	*n* = 32	0.0613 ^1^
Range	0.89–43.00	0.78–42.00	0.75–14.20	
Me	6.51	2.95	3.21	
Neopterin (nmol/L)	*n* = 26	*n* = 18	*n* = 32	0.005 ^1,^*
Range	1.01–43.26	1.22–13.44	1.46–39.23	^a–c^ 0.037 ^2,^*
Me	3.78 ^a^	3.82 ^b^	11.55 ^c^	^b–c^ 0.010 ^2,^*
Amylase (U/mL)	*n* = 26	*n* = 17	*n* = 30	0.125 ^1^
Range	17.35–264.00	10.63–335.40	17.01–523.09	
Me	63.15	97.80	115.00	

Legend: ^1^ The Kruskal–Wallis test, ^2^ post hoc test, * α = 0.05, Me—median, indication of significant differences between the specified parameters: ^a–c^, ^b–c^, *n*—the number of saliva samples in which the test was performed (in some cases, the amount of saliva collected was not sufficient to perform the test).

**Table 8:** Comparison of buffer capacity of stimulated saliva from study groups and the control group.

Stimulated Saliva (SS)	Study Groups: Oncological Patients Treated with BPs			
	with MRONJ (Group I) *n* = 29 (100%)	without MRONJ (Group II) *n* = 20 (100%)	Control Group *n* = 32 (100%)	*p*-Value
Low buffer capacity	4 (13.8%)	4 (20.0%)	2 (6.3%)	0.152 ^1^
Medium buffer capacity	8 (27.6%)	3 (15.0%)	4 (12.5%)	
High buffer capacity	17 (58.6%)	13 (65.0%)	26 (81.3%)	

Legend: ^1^ Chi-square test.

**Table 9:** Comparison of *S. mutans* and *Lactobacillus* spp. load in stimulated saliva from study groups and the control group.

Stimulated Saliva (SS)		Study Groups: Oncological Patients Treated with BPs			
		with MRONJ (Group I) *n* = 29 (100%)	without MRONJ (Group II) *n* = 20 (100%)	Control Group *n* = 32 (100%)	*p*-Value
*S. mutans*	<10^5^	19 (65.5%) ^a^	17 (89.5%)	29 (96.7%) ^c^	^a–b^ 0.061 ^1^ ^a–c^ 0.002 ^1,^* ^b–c^ 0.306 ^1^
	>10^5^	10 (34.5%) ^a^	2 (10.5%)	1 (3.3%) ^c^	
*Lactobacillus* spp.	<10^5^	14 (48.3%) ^a^	14 (73.7%) ^b^	29 (93.5%) ^c^	^a–b^ 0.081^1^ ^a–c^ <0.001 ^1,^* ^b–c^ 0.050 ^1,^*
	>10^5^	15 (51.7%) ^a^	5 (26.3%) ^b^	2 (6.5%) ^c^	

Legend: ^1^ Chi-square test, * α = 0.05, indication of significant differences between the specified parameters: ^a–b^, ^a–c^, ^b–c^.

## Data Availability

The data presented in this study are available on request from the corresponding author. The data are not publicly available due to privacy restrictions.
